# General joint hypermobility in temporomandibular joint disease; clinical characteristics, biomarkers, and surgical aspects

**DOI:** 10.1016/j.heliyon.2023.e23051

**Published:** 2023-11-30

**Authors:** Mattias Ulmner, Rachael Sugars, Aron Naimi-Akbar, Janne Elin Reseland, Bodil Lund

**Affiliations:** a Medical Unit of Plastic Surgery and Oral and Maxillofacial Surgery, Karolinska University Hospital, 171 76, Stockholm, Sweden; b Department of Dental Medicine, Karolinska Institutet, 171 77, Stockholm, Sweden; c Health Technology Assessment-Odontology, Malmö University, 205 06, Malmö, Sweden; d Department of Biomaterials, Institute of Clinical Dentistry, Faculty of Dentistry, University of Oslo, 0317, Oslo, Norway

**Keywords:** Temporomandibular joint, Temporomandibular joint disorders, Arthroscopy, Joint diseases, Extracellular matrix proteins, Proteins, Synovial membrane

## Abstract

**Objectives:**

This study aimed at identifying biomarkers in the temporomandibular joint (TMJ) synovial tissue analysing 28 extra cellular matrix proteins in TMJ diseased patients, classified with either general joint hypermobility (GJH) or normal joint mobility (NJM), and to compile clinical and protein characterisation to reveal potential surgical predictive factors.

**Study design:**

A prospective observational cohort study including 97 consecutive patients scheduled for TMJ surgery was performed. Joint mobility and several other predefined clinical variables were recorded. Synovial tissue was harvested during surgery followed by examination using multi-analytic profiling. A multivariate quantile regression model was used for analysis purposes.

**Results:**

The GJH/NJM ratio was 2:5. The GJH cohort were younger (*P* = 0.001) and more likely to be women (*P* = 0.026) compared to the NJM cohort. None of the protein concentrations could be correlated to joint mobility in the multivariate regression model, but often to the variable TMJ diagnosis. The surgical outcome after the six-month follow-up were equal between GJH and NJM patients.

**Conclusions:**

GJH was more common in the study cohort compared to general population frequencies, but GJH was not a negative factor for surgical outcome. Young age and female gender correlated to GJH. No TMJ biomarkers were GJH specific, and the results suggested that TMJ diagnosis more strongly correlated to the protein profile compared to GJH and the other investigated variables.

## 1 Introduction

A joint with an exaggerated range of motion is defined to be hypermobile. An individual with several hypermobile joints might be characterised as having generalised joint hypermobility (GJH) with inherent laxity and connective tissue fragility [1]. Thus, GJH is descriptive and not a diagnosis. A person with GJH with associated symptoms from joints and connective tissues might be diagnosed with benign joint hypermobility syndrome (BJHS) according to the revised Brighton criteria [2]. GJH, together with other clinical complications, such as mitral valve prolapse, irritable bowel syndrome, chronic pain, anxiety disorders, could suggest the connective tissue disorders Marfan syndrome or Ehlers-Danlos syndrome (EDS) [1]. In the general adult European population between 5 and 15% have been described with GJH [[3], [4], [5], [6], [7]]. Younger age and female gender have also been identified with higher frequencies of GJH, as high as 3.5:1 in female/male ratio [[6], [7], [8]].

GJH has been found to correlate to temporomandibular joint (TMJ) disc displacement (DD) and other TMJ disorders, which might suggest GJH as a factor in TMJ disorder development [[9], [10], [11]]. DD of the TMJ is a relatively common disorder affecting up to 25% of the adult population with various degrees of symptoms [[12], [13], [14]]. Non-specified TMJ disorders, including DD, osteoarthritis, arthralgia etc have a prevalence of 31% [14]. A hypermobile TMJ is thought to wear more extensively because of the exaggerated range of motion, which induces mechanical overload [15]. Another theory is that genetic alterations, coding for several collagen subtypes and extracellular matrix (ECM) proteins have altered synthesis and degradation processes, and therefore predispose for a faster joint deterioration [1,15]. On the other hand, there are studies where no correlation between GJH and TMJ disorders have been found [16,17].

To measure and classify GJH, Carter and Wilkinson proposed a scoring system that was later modified by Beighton et al. [18,19]. Peripheral joints are tested for signs of hypermobility, with a score of “1” for positive signs and “0” for negative signs [19]. The test includes passive dorsiflexion and hyperextension of the fifth MCP joint beyond 90° bilateral, passive apposition of the thumb to the flexor aspect of the forearm bilateral, passive hyperextension of the elbow beyond 10° bilateral, passive hyperextension of the knee beyond 10° bilateral, and active forward flexion of the trunk with the knees fully extended so that the palms of the hands rest flat on the floor. A maximum score of 9 is the result of positive signs of hypermobility regarding all tested joints, and the score 0 indicates no hypermobile joints [19]. The Beighton score is still in use and has gained general acceptance, alone or adopted in the earlier mentioned Brighton criteria [1,2,8]. Hypermobility of the TMJ is difficult to measure in a reliable way and uniformly accepted measures are still lacking,even when using radiology and measuring condylar translation [20,21].

To our knowledge no studies have explored the TMJ ECM composition including collagens, proteoglycans and other matrix proteins, cytokines or enzymes involved in the synthesis and/or remodelling of these proteins, in patients with GJH. The aim of the present study was to investigate a patient cohort with diagnosed TMJ disorder, where patients with GJH were compared to patients with normal joint mobility (NJM) with respect to different demographic and clinical aspects, as well as to perform tissue analyses of various ECM proteins or proteins associated with ECM turnover, harvested from the posterior disc attachment.

The hypothesis was that patients with GJH (Beighton score cut-off ≥ 4) were more prone to an inferior surgical outcome compared to patients with NJM (Beighton score ≤ 3), which would be reflected in divergent ECM protein profiles. Secondary hypotheses were that patients with GJH would be overrepresented in the TMJ disorder cohort admitted to and surgically intervened at our clinic, and that ECM proteins in GJH patients would have different levels compared to NJM patients, making biomarker profiling possible.

## 2 Materials and method

### 2.1 Study design and cohort

A prospective clinical observational trial following the Helsinki Declaration guidelines was performed at Karolinska University Hospital, Stockholm. Ethical approval from the Swedish Ethical Review Authority was granted (2014/622-31/1). Written informed consent was mandatory before inclusion. A power calculation was performed based on the success rate after discectomy of previous studies, the occurrence of GJH in the population of patients offered TMJ discectomy, and the hypothesis that GJH will increase failure rates with 40% [10,22]. The number of participants required were 21 in the GJH group and 68 in the NJM group. An estimated drop-out rate of 10% resulted in 24 GJH patients and 75 NJM patients. This ensured a power of 0.8 and a *P*-value of 0.05.

Consecutive adult patients referred to the Medical Unit of Plastic Surgery and Oral and Maxillofacial Surgery at Karolinska University Hospital, Stockholm, with a diagnosis of DD with or without reduction (DDwR or DDwoR respectively) or degenerative joint disease (DJD), as well as patients with a local and general diagnosis of chronic inflammatory arthritis (CIA) were considered for inclusion [23,24]. The later patient group had to have a prior diagnosis of rheumatoid arthritis, psoriatic arthritis, spondylarthritis, or likewise, set by a rheumatologist and local findings in accordance with specific arthritis criteria [23]. The department’s criteria for surgery based on the stipulated diagnoses, followed the Swedish National Board of Health and Welfare recommendations, and was used as inclusion criteria [25]. Thus, non-invasive treatments, such as physiotherapy, orthotic splint, pharmacological treatment for a period of at least three months was a prerequisite for considering TMJ surgery. Both TMJ pain on function and TMJ disability had to be graded as ≥4 on a visual analogue scale (VAS). Patients with DDwoR had to have a maximum interincisal opening (MIO) of ≤35 mm to be considered for surgery. Further, DDwR patients had to have reciprocal clicking and intermediate catching of the disc in conjunction with severe pain and functional limitation. Exclusion criteria were patients younger than 18 years, prior open joint surgery, or patients not able to give informed consent.

### 2.2 Clinical investigation and tissue sample collection

Four calibrated assessors examined all patients referred to the unit and calibration was initially performed and then at regular six-month intervals. Data concerning demographics, anamnesis and clinical examination variables were gathered. Diagnostic criteria/temporomandibular joint disease (DC/TMD) instructions were used regarding the clinical examination [24]. Four different patient-assessed variables were used, TMJ pain on mandibular function (TMJ pain), TMJ dietary and functional limitations (TMJ disability), psychosocial affection due to TMJ disorder (TMJ psychosocial), and overall bodily pain not including TMJ pain (global pain). A VAS, graded in centimetres, with the endpoints 0 and 10 was used as the assessment instrument. Zero represented no pain/no disability/no psychosocial affection and 10 represented worst imaginable pain/disability/psychosocial affection respectively [26,27].

Participating patients fulfilling above stipulated criteria for surgery, were scheduled for arthroscopic lysis and lavage if diagnosed with DDwoR, DJD, or CIA, and patients diagnosed with DDwR were scheduled for discectomy. Both interventions were performed under general anaesthesia [22,28]. Synovial tissue samples were in all cases taken from the posterior bilaminar zone in the superior joint compartment. In case of bilateral surgery, the tissue sample was taken from the TMJ specified by the patient as the worst affected. Arthroscopy sampling with biopsy forceps (Karl Storz SE & Co, Tuttlingen, Germany) were performed under direct visualisation to obtain a representative sample. The resulting synovial samples were approximately 4 mm^2^. The biopsies were immediately placed in RNAlater (ThermoFisher Scientific, Waltham, MA, USA) and refrigerated for one day. Thereafter, RNAlater was disgorged, and the samples stored at −80 °C until protein extraction.

### 2.3 Assessment of surgical outcome

Surgical outcome was evaluated according to four different parameters, three based on the patient’s own perceptions and one based on clinical measurements, at the six-month follow up visit postoperatively. The three subjective variables were TMJ pain, TMJ disability, and TMJ psychosocial, with a cut-off value of VAS ≤3 (or diminished by at least 40% compared to preoperative ratings) and the objective variable was MIO with a cut-off of ≥35 mm (or increased more than 40% compared to preoperative measurement). A successful treatment was defined as fulfilling all four parameters. The good outcome category was characterised based on fulfilling one or two of the three subjective variables and the objective measure. The intermediate outcome should not fulfil the above stated criteria and the deteriorated outcome described a clear deteriorated patient compared to the preoperative appearance.

#### 2.3.1 Synovial tissue analysis

Each sample was individually disrupted by grinding in liquid nitrogen. Proteins were extracted from the tissue using an ice-cold NP40 cell lysis buffer (ThermoFisher Scientific) with a ratio of 50 μL buffer per 10 mg of tissue [27,29]. The resulting mixture was centrifuged for 10 min at 20,000 g and 4 °C. The total protein concentration in the supernatant was analysed using the Qubit Protein Assay Kit and the Qubit Fluorometer (ThermoFisher Scientific). A variety of different known ECM proteins or proteins involved in ECM turnover were chosen and three different magnetic bead panels were then used to analyse specific protein concentrations: Human MMP Magnetic Bead Panel 2 (HMMP2MAG-55K [Merck Millipore, Burlington, MA, USA]) for matrix metalloproteinases (MMP) 1, 2, 7, 9, and 10, Human TIMP Magnetic Bead Panel 2 (HTMP2MAG-54K [Merck Millipore]) for tissue inhibitors of metalloproteinases (TIMP) 1, 2, 3, and 4, and Human Magnetic Luminex Assay 20 plex (LXSAHM-20 [R&D systems, Bio-Techne Corp., Minneapolis, MN, USA]) custom produced for a disintegrin and metalloproteinase with a thrombospondin type 1 motif member 13 (ADAMTS13), aggrecan, collagen types I α1 and IV α1, fibroblast activation protein α (FAP-α), fibronectin, hepatocyte growth factor receptor (HGF-r), intercellular adhesion molecule 1 (ICAM 1), lumican, neural cell adhesion molecule-1 (NCAM-1), osteoprotegerin (OPG), osteonectin, syndecan-1, and -4, tenascin C, and triggering receptor expressed on myeloid cells 1 (TREM1). A Luminex 200 system (Luminex, Austin, TX, USA) and xPONENT 3.1 software (Luminex) were used to analyse the panels and the resulting data.

### 2.4 Statistical analysis

The software Stata version 15 SE (StataCorp, Collage Station, TX, USA) and IBM SPSS version 28.0 (IBM Corp, Armonk, NY, USA) were used for analyses. Demographic and clinical data were summarised as mean ± standard deviation for all continuous data, and as number and percentage for bivariate data. Student’s T-test were used to analyse continuous data on patient characteristics and Chi-Square test for the categorical data. The surgical outcome-groups were merged to create a dichotomous outcome, where patients with a successful and good outcome were compiled and the intermediate and deteriorated patients constituted the other group. The concentration of specified proteins (pg/mL) was used in the statistical analyses. None of the protein’s concentrations were normally distributed and therefore median values were used for descriptive and comparative statistics. Quantile regression based on 20 bootstrap samples was performed to compare GJH and NJM protein concentrations. NJM protein concentrations were set as reference. To adjust for possible confounding factors a multivariate quantile regression analysis was also performed, including the parameters age, gender, and diagnosis. Age was divided into three categories, age 18–29 which was also set as the reference, age 30–49, and age ≥50. Gender had men as the reference and diagnosis had DDwR as reference, also including the diagnoses DDwoR, DJD, and CIA. A *P-*value of ≤0.05 was regarded as significant.

## 3 Results

### 3.1 Included patients

**Fig. 1:**
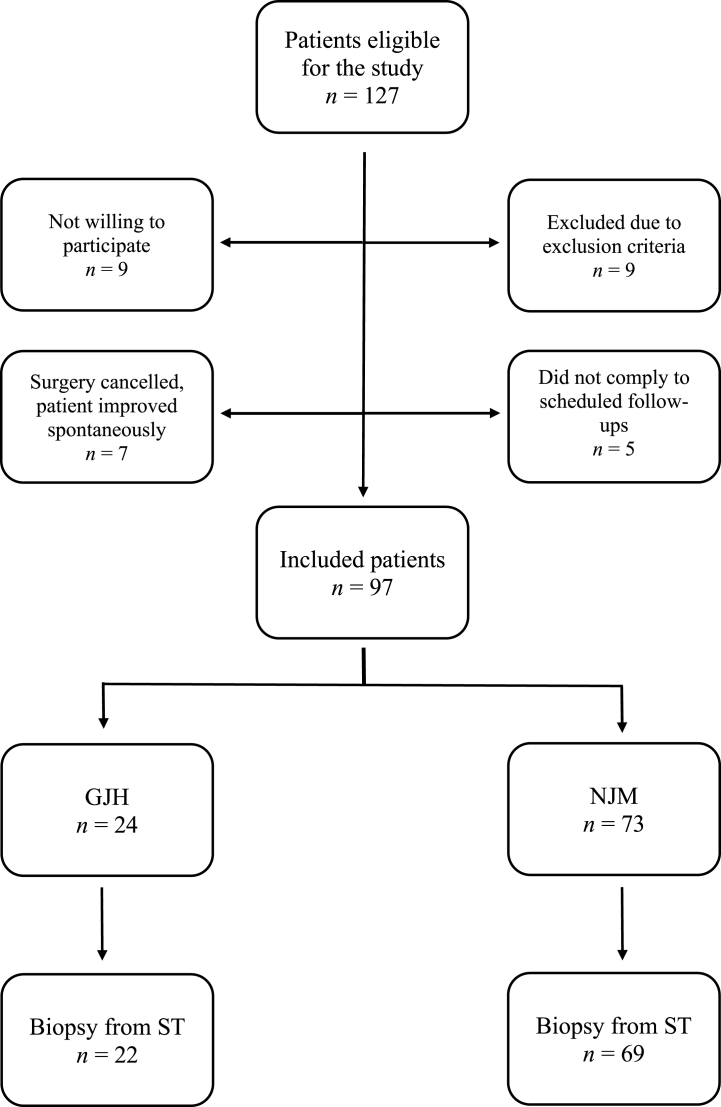
Flow chart illustrating the included and excluded patients with either normal joint mobility (NJM) or general joint hypermobility (GJH), as well as harvested synovial tissue (ST) in the respective cohort.

From a total of 127 eligible patients examined from December 2014 to January 2017, 97 were included in the study (Fig. 1). The 30 patients not included had an average age of 39.3 (±14.6), the women/men ratio were 5:1 and the GJH/NJM ratio were 1:2 (missing GJH/NJM data on 15 patients). Considering these demographic parameters, no significant differences were apparent compared to the included patients (age, *P* = 0.321; gender, *P* = 0.765; joint mobility, *P* = 0.530) and therefore the investigated group might be considered as representative.

The study patients had a GJH/NJM proportion of 24/73 (Table 1). Thereby, the GJH patients constituted 25% of the cohort, which was higher than the general reported epidemiologic figures [[3], [4], [5], [6]]. There were significantly more women in the GJH group (*P* = 0.026) (Table 1), in fact all the GJH patients were women. Of the GJH patients only one had a connective tissue disorder, in this case EDS. Preoperative MIO in the GJH group was higher (36.9 mm) compared to the NJM group (33.2 mm) but the difference was not significant (*P* = 0.105). The patients age differed significantly between groups (*P* = 0.001) with the NJM patients being considerably older (Table 1). Only minor, non-significant, differences were found between groups comparing medical status and history. When analysing preoperative clinical and anamnestic variables only TMJ disability was found to be significantly higher in GJH patients (*P* = 0.038) (Table 1).

**Table 1:** Patient characteristics at preoperative registration including demographic, medical, clinical, and anamnestic data.

	NJM					GJH				
	DDwR	DDwoR	DJD	CIA	*Total*	DDwR	DDwoR	DJD	CIA	*Total*
**Demography**										
Age (years), mean (SD)	40.1 (10.9)	46.7 (16.4)	55.0 (20.7)	40.5 (13.6)	*45.5 (16.1)*	33.8 (13.4)	34.6 (11.9)	36.7 (15.3)	29.8 (5.9)	*33.8 (11.9)*
Gender ratio (M/W), *n*	5/8	7/29	0/10	1/13	*13/60*	0/6	0/11	0/3	0/4	*0/24*
Duration (mts), mean (SD)	49.7 (45.0)	19.7 (24.3)	34.7 (54.0)	33.2 (36.9)	*29.8 (37.1)*	30.0 (21.2)	22.3 (23.8)	19.7 (5.1)	79.5 (46.8)	*33.7 (33.9)*
**Medical history, *n* (%)**										
Healthy	6 (46.2)	14 (38.9)	1 (10.0)	0	*21 (28,8)*	4 (66.7)	4 (36.4)	0	0	*8 (33.3)*
Psychiatric disorder	3 (23.1)	10 (27.8)	1 (10.0)	2 (14.3)	*16 (21.9)*	1 (16.7)	5 (45.5)	0	1 (25.0)	*7 (29.2)*
Neuropsychiatric disorder	1 (7.7)	1 (2.8)	0	1 (7.1)	*3 (4.1)*	0	0	0	0	*0*
Autoimmune disease	0	0	0	14 (100.0)	*14 (19.2)*	0	0	1 (33.3)	4 (100.0)	*6 (25.0)*
Metabolic disease	2 (15.4)	7 (19.4)	1 (10.0)	0	*10 (13.7)*	0	0	1 (33.3)	0	*1 (4.2)*
Other disease	4 (30.8)	17 (47.2)	9 (90.0)	7 (50.0)	*37 (50.7)*	1 (16.7)	6 (54.5)	1 (33.3)	3 (75.0)	*11 (45.8)*
**Clinical and anamnestic variables, mean (SD)**										
MIO (mm)	42.4 (9.7)	28.8 (5.1)	40.0 (5.8)	30.9 (7.5)	*33.2 (8.7)*	47.5 (10.3)	30.7 (3.5)	44.7 (5.0)	32.0 (5.4)	*36.9 (9.7)*
TMJ pain (VAS)	4.3 (2.4)	5.5 (2.4)	6.4 (2.5)	6.0 (2.1)	*5.5 (2.4)*	3.8 (3.1)	5.8 (2.7)	6.3 (0.6)	6.9 (1.3)	*5.5 (2.6)*
TMJ disability (VAS)	5.5 (1.9)	6.1 (1.8)	5.7 (2.3)	5.6 (1.8)	*5.8 (1.9)*	7.3 (1.9)	6.8 (1.0)	5.7 (2.5)	6.6 (2.1)	*6.7 (1.6)*
TMJ psychosocial (VAS)	4.9 (3.2)	3.4 (2.8)	6.0 (3.7)	5.7 (2.5)	*4.5 (3.1)*	5.1 (3.7)	4.1 (2.8)	5.5 (5.0)	5.8 (1.9)	*4.8 (2.8)*
Global pain (VAS)	3.1 (3.4)	2.9 (3.2)	4.7 (2.8)	4.7 (3.1)	*3.5 (3.2)*	3.6 (2.9)	3.4 (2.9)	4.7 (2.3)	5.3 (2.1)	*3.9 (2.6)*

CIA, chronic inflammatory arthritis; DDwoR, disc displacement without reduction; DDwR, disc displacement with reduction; DJD, degenerative joint disease; GJH, general joint mobility; M, men; MIO, maximum interincisal opening; mm, millimetre; mts, months; *n*, number; NJM, normal joint mobility; SD, standard deviation; TMJ, temporomandibular joint; VAS, visual analogue scale; W, women.

### 3.2 Surgical outcome

Comparison of the surgical outcome in the GJH cohort to the outcome in the NJM cohort found a non-significant difference (*P* = 0.911) (Table 2). No analyses of the different TMJ diagnosis groups were performed due to the small patient groups, especially in the GJH cohort. The sole EDS patient had a good surgical outcome based on the predefined outcome measures.

**Table 2:** Outcome of TMJ surgery at 6 months with respect to joint mobility and TMJ diagnosis.

Joint mobility	TMJ diagnosis	Outcome variables, mean (SD)			Outcome grading, n (%)			
		MIO	TMJ pain	TMJ disability	Successful	Good	Intermediate	Deteriorated
GJH	Total	41.9 (5.6)	3.1 (2.9)	3.1 (2.4)	12 (50)	7 (29)	4 (17)	1 (4)
	DDwR	45.2 (4.2)	2.7 (3.1)	2.9 (3.2)	4 (67)	0	2 (33)	0
	DDwoR	39.7 (5.7)	2.7 (2.9)	3.2 (2.1)	6 (55)	3 (27)	2 (18)	0
	DJD	45.3 (2.5)	3.0 (2.0)	2.2 (0.3)	0	3 (100)	0	0
	CIA	40.5 (6.5)	5.0 (3.2)	4.1 (3.4)	2 (50)	1 (25)	0	1 (25)
NJM	Total	40.0 (6.9)	2.6 (2.4)	3.0 (2.5)	43 (59)	14 (19)	16 (22)	0
	DDwR	43.5 (7.6)	1.4 (1.2)	2.3 (1.6)	9 (69)	3 (23)	1 (8)	0
	DDwoR	39.4 (6.6)	2.9 (2.7)	3.2 (2.8)	19 (53)	10 (28)	7 (19)	0
	DJD	39.9 (5.5)	2.6 (2.5)	2.7 (2.4)	8 (80)	1 (10)	1 (10)	0
	CIA	38.5 (8.0)	2.9 (2.5)	3.4 (2.6)	7 (50)	0	7 (50)	0

CIA, chronic inflammatory arthritis; DDwoR, disc displacement without reduction; DDwR, disc displacement with reduction; DJD, degenerative joint disease; GJH, general joint hypermobility; MIO, maximum interincisal opening; *n*, number; NJM, normal joint mobility; TMJ, temporomandibular joint.

**Fig. 2:**
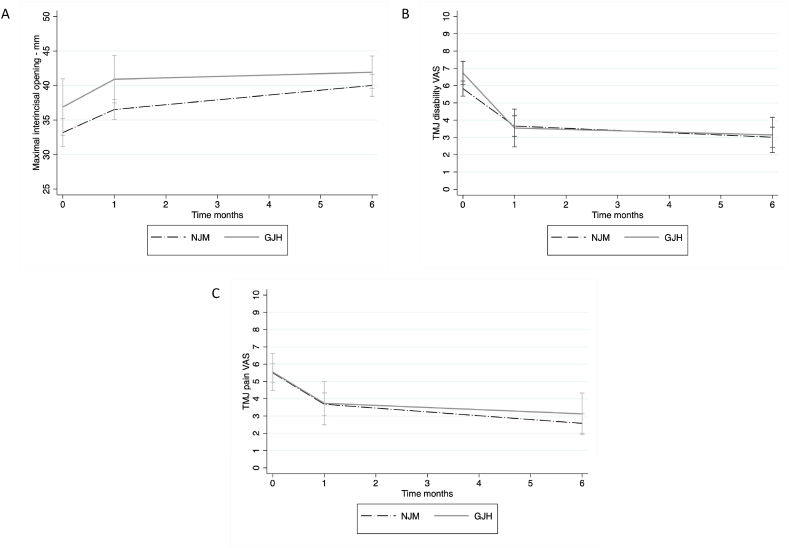
Longitudinal data on patient-specific variables. Diagrams showing the mean values of maximal interincisal opening (MIO) (A), temporomandibular joint (TMJ) disability (B), and TMJ pain (C) from preoperative registration to the six-months follow-up. Whiskers show lower and upper quartiles. GJH, general joint hypermobility; NJM, normal joint mobility; VAS, visual analogue scale.

Correlation of the total cohorts’ preoperative values to the 6-month postoperative values gave mean differences and p-values as follows, MIO +6.4 mm, *P* < 0.001; TMJ pain −2,8 (VAS), *P* < 0.001; TMJ disability, −3.0 (VAS), *P* < 0.001; TMJ psychosocial, −2.1 (VAS), *P* < 0.001; global pain, −0.6 (VAS), *P* = 0.41. Dividing the total cohort into sub-groups NJM and GJH showed that there were no significant differences in the 6-month outcome regarding the variables MIO (*P* = 0.221), TMJ pain (*P* = 0.268), TMJ disability (*P* = 0.821), TMJ psychosocial (*P* = 0.191), and global pain (*P* = 0.362). The longitudinal values of the outcome variables MIO, TMJ pain, and TMJ disability clearly showed that the most pronounced effect of the treatment was observed one month postoperatively, whereafter the improvement continued but was more modest (Fig. 2).

#### 3.2.1 Protein content of synovial tissue samples

The concentrations of the various proteins isolated from synovial tissues are given in Table 3. When comparing the analysed concentrations of ECM proteins and proteins associated with ECM in patients with either GJH or NJM in a univariate quantile regression, FAP-α, OPG, and TIMP-3 showed significantly different protein levels (Table 3). GJH patients had higher levels of FAP-α and OPG, whereas NJM patients had larger amounts of TIMP-3.

**Table 3:** Univariate quantile regression comparing TMJ synovial tissue protein concentrations in patients with GJH to patients with NJM.

	GJH/NJM (*n*)	Concentration (pg/mL)		Quantile regression			
Protein		Min-max	Median	Coef.	*P*	95% CI	
ADAMTS13	21	3.4 × 10^4^–1.6 × 10^6^	1.7 × 10^5^	−6.8 × 10^4^	0.587	−3.1 × 10^5^	1.8 × 10^5^
	63	2.4 × 10^4^–2.3 × 10^6^	2.3 × 10^5^				
Aggrecan	22	1851.0–37923.4	7709.7	1385.9	0.771	−8027.1	10798.9
	69	192.8–2.5 × 10^5^	7826.6				
BMP-2	22	66.4–2426.6	392.2	144.0	0.294	−127.0	415.1
	69	1.7–2397.8	283.2				
BMP-4	22	70.8–1457.3	142.3	−139.7	0.216	−362.5	83.2
	69	2.6–2125.2	403.1				
BMP-9	13	0.3–1451.8	107.8	−65.9	0.704	−413.4	281.6
	30	0.4–1028.4	168.2				
Collagen 1 α1	22	966.9–1.3 × 10^5^	26902.9	9971.7	0.116	−2525.2	22468.5
	69	0.9–3.3 × 10^5^	19997.2				
Collagen 4 α1	22	1805.9–61143.0	10418.8	282.9	0.945	−7903.3	8469.0
	69	6.0–96130.7	11052.2				
FAP-α	22	71.6–71965.3	11727.4	7784.3	0.019	1300.4	14268.2
	69	0.9–1.1 × 10^5^	5570.5				
Fibronectin	22	1.3 × 10^4^-1.2 × 10^7^	2.1 × 10^6^	5.7 × 10^5^	0.191	−2.9 × 10^5^	1.4 × 10^6^
	69	775.2–2.1 × 10^7^	1.5 × 10^6^				
HGF-r	22	444.0–7781.1	3359.1	1771.7	0.065	−109.1	3652.5
	69	8.4–19226.3	1770.1				
ICAM-1	22	1.9 × 10^5^-4.1 × 10^6^	8.1 × 10^5^	2.1 × 10^5^	0.524	−4.4 × 10^5^	8.5 × 10^5^
	69	1436.8–4.2 × 10^6^	6.8 × 10^5^				
Lumican	22	6.5 × 10^5^-1.3 × 10^7^	2.2 × 10^6^	−4.2 × 10^5^	0.687	−2.5 × 10^6^	1.6 × 10^6^
	69	7.8 × 10^4^-2.1 × 10^7^	2.8 × 10^6^				
MMP-1	16	41.0–868.9	408.0	176.3	0.095	−31.6	384.2
	47	30.3–1278.1	205.8				
MMP-2	22	745.2–2.6 × 10^5^	42456.0	33010.1	0.087	−4952.2	70972.4
	68	1475.4–3.0 × 10^5^	18247.4				
MMP-7	14	3292.2–47031.2	10286.1	700.5	0.937	−16867.0	18268.0
	46	3292.2–68587.1	10973.9				
MMP-9	22	74.8–150986.6	14677.3	9326.4	0.054	−179.0	18831.7
	68	9.2–330309.8	5391.2				
MMP-10	21	27.4–3100.0	472.2	165.7	0.502	−323.1	654.5
	65	2.1–5651.2	306.5				
NCAM-1	22	5976.1–66123.5	12492.2	−2508.6	0.493	−9742.1	4725.0
	69	159.0–96430.0	15018.4				
OPG	22	321.4–202633.2	4811.3	2283.7	0.042	0.0	4487.6
	69	5.889–61073.1	2527.7				
Osteonectin	22	6537.6–1.1 × 10^6^	1.6 × 10^5^	56379.9	0.271	−44669.8	1.6 × 10^5^
	69	471.2–2.2 × 10^6^	1.5 × 10^5^				
Syndecan-1	22	982.4–65257.9	3909.9	1576.9	0.431	−2387.3	5541.0
	69	11.3–14303.0	2753.9				
Syndecan-4	22	217.7–5546.4	704.6	207.5	0.191	−105.2	520.1
	69	0 2478.6	518.5				
Tenascin C	22	566.5–1.4 × 10^5^	21594.6	12341.5	0.082	−1617.5	26300.5
	69	21.9–2.2 × 10^5^	11717.4				
TIMP-1	22	335.8–39833.7	10264.4	2502.0	0.540	−5570.7	10574.7
	68	378.9–43297.0	8156.5				
TIMP-2	22	758.3–52381.6	12841.5	−800.2	0.733	−5445.8	3845.5
	68	1109.7–76750.1	13592.1				
TIMP-3	22	279.6–13111.8	2283.0	−1159.8	0.043	−2284.2	−35.3
	68	151.0–31820.0	3419.0				
TIMP-4	22	5.2–806.8	76.2	−2.5	0.902	−43.1	38.1
	68	1.6–438.1	79.8				
TREM1	20	46.2–2328.3	776.6	88.1	0.749	−458.0	634.1
	63	3.4–5264.0	697.9				

CI, confidence interval; coef., coefficient; GJH, general joint hypermobility; mL, millilitre; *n*, number; NJM, normal joint mobility; pg, picogram.

In the multivariate regression analysis, none of the proteins correlated to joint mobility, when including sex, age, and TMJ diagnosis, in addition to GJH/NJM (Supplementary Table SI). However, HGF-r was close, showing a correlation to joint mobility (coef., 1337.2; *P* = 0.051). TMJ diagnosis, and especially DDwoR in relation to DDwR, showed a strong correlation to changes in the protein profiles in 20 of the 28 proteins analysed (Supplementary Table S1). Five proteins; osteonectin, OPG, syndecan-4, tenascin C, and TIMP-4, showed no significant correlations to any of the analysed variables in the multivariate computations. It is noteworthy that OPG, significantly correlated to joint mobility in the univariate analysis, had no strong correlation to any of the variables in the multivariate analysis. MMP-10 showed a significant difference between age group 18–29 and age group 30–49 (coef., 234.7; *P* = 0.019). Collagen type 1 α1 also significantly differed but between age 18–29 and age ≥50 (coef., 19405.8; *P* = 0.004). TIMP-3 was the only protein that showed a significant difference in relation to sex (coef., 2620.0; *P* = 0.038).

A subgroup analyses on the age group 18–29 (*n* = 30; women, *n* = 26; patients with synovial tissue sample, *n* = 28) was performed. This was due to reports on decreasing Beighton score due to increasing age, which might result in an under-registration of GJH in elderly patients, and thereby skew the analyses [[6], [7], [8]]. The multivariate regression analyses did not reveal any differences between NJM and GJH patients concerning any of the 28 investigated ECM proteins in the age group 18–28 either (Supplementary Table SII). These analyses also showed that TMJ diagnosis primarily correlated to deviating protein levels.

Another aim was to analyse ECM protein profiles in relation to GJH/NJM and outcome of TMJ surgery. However, since no statistically significant differences were found between GJH and NJM no analyses of correlation between surgical outcome and ECM protein concentration were made.

## 4 Discussion

GJH has been proposed to be a negative and potentially a causative factor in developing TMJ disease [[9], [10], [11]]. This would suggest that patients with TMJ disease, such as DD, would also be classified with GJH to a greater extent compared to a normal population. This has been shown in a study by Hirsch et al. but refuted by others [7,16,30]. In the current study as many as 25% had GJH out of all patients attending the department with the diagnoses DDwR, DDwoR, DJD, or CIA, and with indications for surgery. This was a higher number compared to the somewhat uncertain figures of 5–15% described for European adult populations [[3], [4], [5], [6]]. This implies that TMJ-diseased patients to a higher extent might be categorised with GJH. An age and sex matched non-TMJ diseased control group might have verified the higher prevalence, though. GJH’s role in TMJ disease is still to be verified.

The hypothesis stated that GJH patients would have a less good outcome of surgery compared to NJM patients owing to an altered ECM composition negatively affecting the surgical result, however this could not be confirmed. Both patient groups showed equally good surgical outcomes after six months follow up. According to this, using GJH classified with the Beighton score as a surgical outcome predictor, proved to be inadequate. Further, after analysing synovial tissue biopsies from the patients, there were no significant differences in ECM proteins and ECM-associated proteins that could be related to joint mobility in the multivariate analyses. Differences in protein level often strongly correlated to TMJ diagnosis, and in some cases was dependent on age or sex, but never correlated to joint mobility. Earlier theories proposing a hypermobile joint would wear more extensively because of the extended range of motion or that genetic alterations affecting ECM proteins would result in altered synthesis and/or degradation processes could not be verified in this study [1,15]. The TMJ diagnosis impact on the protein profiles might be associated with inflammation, osteoarthritis and/or fibrotic changes in the TMJ. Since the included diagnoses roughly displayed increased inflammation, degradation, and fibrosis, with DDwR in one end of the disease spectrum and CIA in the other, diagnosis might then be a good marker for ECM protein concentration differences. Since an age difference has been shown in this material with GJH patients being significantly younger when scheduled for surgery, one might speculate that the joint wear in GJH patients is more aggressive compared to NJM patients. Reflecting over the fact that diagnosis seems to be the most influencing factor regarding concentration differences in these patients, GJH patients might have experienced a faster intra-articular deterioration compared to NJM patients. However, further studies are warranted to reveal any linkage to GJH.

Interestingly, the levels of TIMP-4, OPG, osteonectin, tenascin C, and syndecan-4, were not correlated to any of the variables investigated as might have been anticipated. To exemplify, tenascin C has been shown to be active in post-trauma scar formation, in knock out mice with reduced intra articular TMJ fibrous adhesion [31]. Low concentrations of OPG has been related to increased osteoclastogenesis and increased bone resorption [32]. Furthermore, in an earlier study, we have shown high OPG concentrations to be strongly correlated with longer symptom duration in TMJ disease, suggesting OPG as a time-dependent bone protective factor [33]. The absence of syndecan-4 in knock-out mice, showed a decreased TNF-α expression, which in turn reduced synovial inflammation and increased cartilage thickness [34]. Changes to the proteins profile might be anticipated, especially when TMJ diagnosis appears to strongly correlate to the protein concentration, as supported in earlier work on the characterisation of DDwR contra DDwoR [33]. Potentially, other clinical or biological, yet unknown variables may correlate with the proteins profile and warrant further investigation in the search for TMJ disease aetiopathogeneses.

There is no known link between a classification of GJH and the presentation of local TMJ hypermobility, but there are indications that a greater MIO is related to GJH [7]. A hypothesis might be that the higher the Beighton score the higher the possibility for local TMJ hypermobility. An obstacle to this, would be that to date there are no validated criteria for classifying local TMJ hypermobility, which calls upon further studies on the topic [20,21]. No significant difference between GJH/NJM was detected when analysing MIO in this study. Although, all of the investigated cohort had a TMJ diagnosis, which might have levelled the eventual pre-TMJ diagnosis differences. In a TMJ healthy population increased MIO might have a positive correlation with an elevated Beighton score [7].

Beighton score has been ascertained with good inter- and intra-rater reliability, making the screening reproducible to other researchers and clinicians [5,35]. Nevertheless, the Beighton score has been questioned because the older the patient with increased stiffness, the less scoring the patient gets [6]. In the investigated patient cohort, the mean age of the GJH patients was significantly lower compared to the NJM patients, which might indicate an under-registration of GJH patients in the older included patients. Suggestions have been made to alter the Beighton score cut-off level in accordance with age [6]. Performing a subgroup analysis only on the age group 18–29 to adjust for biased Beighton scoring did not result in any deviating results regarding the protein profiles. In fact, the result in this age group also highlighted that of the investigated variables, TMJ diagnosis seems to be the most important variable correlating with protein concentration.

A limitation of this study was that we had a very small tissue piece from each patient which narrowed the number of proteins that could be analysed. A larger tissue sample was not appropriate to harvest because that might theoretically hamper the result of the provided surgery. Interesting ECM proteins not included in these analyses were collagen III, elastin, laminin, etc. Further studies on proteins and joint mobility are warranted.

In conclusion, there was an over-representation of GJH patients in this cohort. Although, GJH does not seem to be a negative factor influencing the surgical outcome, since both NJM and GJH patients showed equal outcome frequencies. Analysis of selected ECM proteins and proteins associated with ECM did not reveal any differences between the groups NJM/GJH. Thus, it can be suggested that GJH with a Beighton score cut off value of ≥4 cannot be considered as an outcome prognostic marker for TMJ surgery, neither can any of the 28 investigated proteins be regarded as biomarkers in GJH patients with TMJ disease. Instead, TMJ diagnoses seems to correlate strongly to the levels of many ECM proteins.

## Funding

This study was supported by grants from the Swedish Dental Society, 10.13039/501100004047Karolinska Institutet, 10.13039/501100005036University of Bergen and HelseVest funding, 10.13039/501100010586Haukeland University Hospital.

## Data availability statement

The data associated with this study has not been deposited in a publicly available repository, but the data will be made available upon request.

## CRediT authorship contribution statement

**Mattias Ulmner:** Writing – review & editing, Writing – original draft, Visualization, Validation, Methodology, Investigation, Funding acquisition, Formal analysis, Data curation, Conceptualization. **Rachael Sugars:** Writing – review & editing, Supervision, Resources, Project administration, Methodology, Conceptualization. **Aron Naimi-Akbar:** Writing – review & editing, Investigation, Formal analysis, Data curation. **Janne Elin Reseland:** Writing – review & editing, Resources, Investigation. **Bodil Lund:** Writing – review & editing, Supervision, Resources, Project administration, Methodology, Funding acquisition, Conceptualization.

## Declaration of competing interest

The authors declare that they have no known competing financial interests or personal relationships that could have appeared to influence the work reported in this paper.
