# Supplementary material for: General joint hypermobility in temporomandibular joint disease; clinical characteristics, biomarkers, and surgical aspects
Source: Heliyon. 2023 Nov 30;9(12):e23051. doi: 10.1016/j.heliyon.2023.e23051 (PMC10750043; doi:10.1016/j.heliyon.2023.e23051)
Supplement: Multimedia component 1 [file mmc1.docx]

**Supplementary Table SI.** Multivariate quantile regression of ECM protein and ECM-related protein concentrations in patients with GJH or NJM.

| **Protein** | **Variables** | | **Coef.** | ***P*** | | **95% CI** | |
| --- | --- | --- | --- | --- | --- | --- | --- |
|  | ***Reference*** |  |  | |  | **Lower** | **Upper** |
| ADAMTS13 | NJM | GJH | 56913.7 | 0.526 | | -1.2x10^5^ | 2.3x10^5^ |
|  | Age 18-29 | Age 30-49 | 18233.7 | 0.781 | | -1.1x10^5^ | 1.5x10^5^ |
|  |  | Age 50 + | 1.1x10^5^ | 0.359 | | -1.2x10^5^ | 3.3x10^5^ |
|  | Men | Women | -17354.1 | 0.819 | | -1.7x10^5^ | 1.3x10^5^ |
|  | DDwR | DDwoR | 3.6x10^5^ | 0.009 | | 92343.2 | 6.3x10^5^ |
|  |  | DJD | -17962.3 | 0.829 | | -1.8x10^5^ | 1.5x10^5^ |
|  |  | CIA | 32619.6 | 0.592 | | -87996.1 | 1.5x10^5^ |
| Aggrecan | NJM | GJH | 109.7 | 0.945 | | -3047.4 | 3266.9 |
|  | Age 18-29 | Age 30-49 | 1622.8 | 0.465 | | -2771.4 | 6016.9 |
|  |  | Age 50 + | -180.5 | 0.939 | | -4829.8 | 4468.8 |
|  | Men | Women | -267.2 | 0.938 | | -7066.2 | 6531.7 |
|  | DDwR | DDwoR | 4471.9 | 0.041 | | 177.1 | 8766.7 |
|  |  | DJD | 15326.4 | 0.001 | | 6132.7 | 24520.1 |
|  |  | CIA | 31164.6 | 0.044 | | 904.8 | 61424.5 |
| Collagen1 α1 | NJM | GJH | 5201.3 | 0.478 | | -9324.3 | 19727.0 |
|  | Age 18-29 | Age 30-49 | 4150.4 | 0.517 | | -8548.9 | 16849.7 |
|  |  | Age 50 + | 19405.8 | 0.004 | | 6367.0 | 32444.7 |
|  | Men | Women | -361.5 | 0.938 | | -9547.9 | 8824.9 |
|  | DDwR | DDwoR | 14612.5 | 0.063 | | -802.8 | 30027.8 |
|  |  | DJD | -13626.6 | 0.071 | | -28451.3 | 1198.0 |
|  |  | CIA | -9324.4 | 0.057 | | -18918.8 | 269.9 |
| Collagen4 α1 | NJM | GJH | 2261.6 | 0.578 | | -5797.8 | 10321.1 |
|  | Age 18-29 | Age 30-49 | 1533.7 | 0.524 | | -3237.0 | 6304.4 |
|  |  | Age 50 + | 4786.3 | 0.078 | | -539.5 | 10112.1 |
|  | Men | Women | -94.9 | 0.967 | | -4688.7 | 4498.9 |
|  | DDwR | DDwoR | 8872.8 | 0.010 | | 2142.3 | 15603.3 |
|  |  | DJD | -552.7 | 0.908 | | -10082.4 | 8977.0 |
|  |  | CIA | 3942.1 | 0.286 | | -3356.2 | 11240.5 |
| FAP-α | NJM | GJH | 2508.9 | 0.514 | | -5110.6 | 10128.5 |
|  | Age 18-29 | Age 30-49 | 1938.5 | 0.557 | | -4594.5 | 8471.5 |
|  |  | Age 50 + | 4431.2 | 0.242 | | -3053.3 | 11915.7 |
|  | Men | Women | 2163.4 | 0.411 | | -3044.8 | 7371.5 |
|  | DDwR | DDwoR | 10110.4 | 0.018 | | 1780.0 | 18440.9 |
|  |  | DJD | -6280.8 | 0.162 | | -15140.2 | 2578.5 |
|  |  | CIA | -6433.0 | 0.086 | | -13800.4 | 934.4 |
| Fibronectin | NJM | GJH | 5.0x10^5^ | 0.358 | | -5.8x10^5^ | 1.6x10^6^ |
|  | Age 18-29 | Age 30-49 | 1.2x10^6^ | 0.037 | | 75546.2 | 2.3x10^6^ |
|  |  | Age 50 + | 9.0x10^5^ | 0.012 | | 2.0x10^5^ | 1.6x10^6^ |
|  | Men | Women | -3.5x10^5^ | 0.669 | | -2.0x10^6^ | 1.3x10^6^ |
|  | DDwR | DDwoR | -1.1x10^6^ | 0.082 | | -2.4x10^6^ | 1.5x10^5^ |
|  |  | DJD | -2.0x10^6^ | 0.094 | | -4.3x10^6^ | 3.4x10^5^ |
|  |  | CIA | -2.1x10^6^ | 0.013 | | -3.7x10^6^ | -4.5x10^5^ |
| HGF-r | NJM | GJH | 1337.1 | 0.051 | | -3.6 | 2677.8 |
|  | Age 18-29 | Age 30-49 | 827.5 | 0.175 | | -375.5 | 2030.6 |
|  |  | Age 50 + | 1323.6 | 0.160 | | -534.8 | 3182.0 |
|  | Men | Women | 732.8 | 0.400 | | -988.4 | 2453.9 |
|  | DDwR | DDwoR | -1216.7 | 0.069 | | -2531.0 | 97.6 |
|  |  | DJD | -3538.6 | 0.001 | | -5604.2 | -1473.0 |
|  |  | CIA | -3140.4 | 0.001 | | -4983.7 | -1297.0 |
| ICAM-1 | NJM | GJH | 1.8x10^5^ | 0.678 | | -6.7x10^5^ | 1.0x10^6^ |
|  | Age 18-29 | Age 30-49 | 1.2x10^5^ | 0.679 | | -4.4x10^5^ | 6.8x10^5^ |
|  |  | Age 50 + | 5.1x10^5^ | 0.089 | | -79327.8 | 1.1x10^6^ |
|  | Men | Women | -1.1x10^5^ | 0.638 | | -5.6x10^5^ | 3.4x10^5^ |
|  | DDwR | DDwoR | 4.8x10^5^ | 0.038 | | 27543.5 | 9.3x10^5^ |
|  |  | DJD | 60244.4 | 0.846 | | -5.6x10^5^ | 6.8x10^5^ |
|  |  | CIA | 16651.7 | 0.955 | | -5.7x10^5^ | 6.0x10^5^ |
| Lumican | NJM | GJH | -2.3x10^5^ | 0.769 | | -1.8x10^6^ | 1.4x10^6^ |
|  | Age 18-29 | Age 30-49 | 1.8x10^5^ | 0.858 | | -1.8x10^6^ | 2.2x10^6^ |
|  |  | Age 50 + | -99814.6 | 0.938 | | -2.6x10^6^ | 2.4x10^6^ |
|  | Men | Women | -1.6x10^5^ | 0.918 | | -3.1x10^6^ | 2.8x10^6^ |
|  | DDwR | DDwoR | 4.7x10^6^ | 0.001 | | 2.0x10^5^ | 7.3x10^5^ |
|  |  | DJD | 3.1x10^5^ | 0.602 | | -8.8x10^5^ | 1.5x10^6^ |
|  |  | CIA | 1.2x10^6^ | 0.251 | | -8.6x10^5^ | 3.2x10^6^ |
| MMP-1 | NJM | GJH | 119.6 | 0.316 | | -117.2 | 356.5 |
|  | Age 18-29 | Age 30-49 | 49.6 | 0.663 | | -176.8 | 275.9 |
|  |  | Age 50 + | 88.5 | 0.418 | | -128.7 | 305.8 |
|  | Men | Women | 70.3 | 0.290 | | -61.6 | 202.1 |
|  | DDwR | DDwoR | 184.7 | 0.009 | | 47.4 | 321.9 |
|  |  | DJD | 92.1 | 0.721 | | -421.3 | 605.4 |
|  |  | CIA | -105.3 | 0.275 | | -296.7 | 86.1 |
| MMP-2 | NJM | GJH | 25890.3 | 0.087 | | -3842.0 | 55622.5 |
|  | Age 18-29 | Age 30-49 | 6542.9 | 0.523 | | -13735.4 | 26821.2 |
|  |  | Age 50 + | 22434.0 | 0.094 | | -3946.3 | 48814.3 |
|  | Men | Women | 6954.5 | 0.598 | | -19162.8 | 33071.8 |
|  | DDwR | DDwoR | 4194.9 | 0.758 | | -22839.0 | 31228.8 |
|  |  | DJD | -36065.0 | 0.022 | | -66907.3 | -5222.7 |
|  |  | CIA | -29570.5 | 0.030 | | -56224 | -2916.9 |
| MMP-7 | Insufficient data | | | | | | |
|  |  |  |  |  |  |  |  |
| MMP-9 | NJM | GJH | 576.2 | 0.881 | | -7084.9 | 8237.3 |
|  | Age 18-29 | Age 30-49 | 725.2 | 0.862 | | -7575.7 | 9026.0 |
|  |  | Age 50 + | 3218.2 | 0.416 | | -4619.4 | 11055.8 |
|  | Men | Women | 6518.2 | 0.148 | | -2355.8 | 15392.1 |
|  | DDwR | DDwoR | -3012.4 | 0.558 | | -13199.0 | 7174.3 |
|  |  | DJD | -15860.2 | 0.040 | | -30998.1 | -722.3 |
|  |  | CIA | -16480.6 | 0.000 | | -24236.0 | -8725.2 |
| MMP-10 | NJM | GJH | 13.9 | 0.949 | | -422.9 | 450.8 |
|  | Age 18-29 | Age 30-49 | 234.7 | 0.019 | | 38.8 | 430.6 |
|  |  | Age 50 + | 57.7 | 0.715 | | -256.0 | 371.4 |
|  | Men | Women | 204.6 | 0.311 | | -194.5 | 603.7 |
|  | DDwR | DDwoR | 249.0 | 0.152 | | -93.8 | 591.9 |
|  |  | DJD | 75.9 | 0.747 | | -391.7 | 543.5 |
|  |  | CIA | 274.8 | 0.204 | | -152.7 | 702.2 |
| NCAM-1 | NJM | GJH | 3175.7 | 0.344 | | -3459.4 | 9810.8 |
|  | Age 18-29 | Age 30-49 | 1660.7 | 0.647 | | -5524.0 | 8845.5 |
|  |  | Age 50 + | 7156.6 | 0.254 | | -5236.8 | 19550.2 |
|  | Men | Women | 1811.1 | 0.687 | | -7093.8 | 10716.1 |
|  | DDwR | DDwoR | 13085.1 | 0.004 | | 4422.9 | 21747.3 |
|  |  | DJD | -912.7 | 0.832 | | -9423.9 | 7598.6 |
|  |  | CIA | 4861.2 | 0.052 | | -47.0 | 9769.3 |
| OPG | NJM | GJH | 940.1 | 0.520 | | -1953.9 | 3834.0 |
|  | Age 18-29 | Age 30-49 | 543.5 | 0.511 | | -1093.0 | 2180.0 |
|  |  | Age 50 + | -570.3 | 0.600 | | -2727.9 | 1587.2 |
|  | Men | Women | 976.1 | 0.676 | | -3647.5 | 5599.8 |
|  | DDwR | DDwoR | -6968.0 | 0.277 | | -19636.0 | 5700.1 |
|  |  | DJD | -6877.0 | 0.357 | | -21648.6 | 7894.7 |
|  |  | CIA | -9046.4 | 0.164 | | -21866.1 | 3773.2 |
| Osteonectin | NJM | GJH | 50911.7 | 0.260 | | -38463.1 | 1.4x10^5^ |
|  | Age 18-29 | Age 30-49 | 73478.3 | 0.136 | | -23655.7 | 1.7x10^5^ |
|  |  | Age 50 + | 73856.2 | 0.141 | | -25066.6 | 1.7x10^5^ |
|  | Men | Women | 40264.2 | 0.566 | | -98698.7 | 1.8x10^5^ |
|  | DDwR | DDwoR | 20548.8 | 0.767 | | -1.2x10^5^ | 1.6x10^5^ |
|  |  | DJD | -97401.9 | 0.163 | | -2.3x10^5^ | 40179.0 |
|  |  | CIA | -41122.3 | 0.530 | | -1.7x10^5^ | 88479.9 |
| Syndecan-1 | NJM | GJH | 1274.9 | 0.159 | | -509.0 | 3058.7 |
|  | Age 18-29 | Age 30-49 | 69.6 | 0.948 | | -2044.1 | 2183.3 |
|  |  | Age 50 + | 105.7 | 0.924 | | -2080.5 | 2291.9 |
|  | Men | Women | 87.9 | 0.919 | | -1621.7 | 1797.5 |
|  | DDwR | DDwoR | 1933.4 | 0.007 | | 531.7 | 3335.1 |
|  |  | DJD | 1038.9 | 0.471 | | -1814.0 | 3891.7 |
|  |  | CIA | 4897.6 | 0.010 | | 1204.7 | 8590.6 |
| Syndecan-4 | NJM | GJH | 222.9 | 0.153 | | -84.9 | 530.8 |
|  | Age 18-29 | Age 30-49 | 178.9 | 0.203 | | -98.6 | 456.4 |
|  |  | Age 50 + | 212.3 | 0.315 | | -205.0 | 629.7 |
|  | Men | Women | 323.8 | 0.107 | | -71.2 | 718.9 |
|  | DDwR | DDwoR | 41.2 | 0.822 | | -322.7 | 405.1 |
|  |  | DJD | -393.4 | 0.184 | | -976.9 | 190.0 |
|  |  | CIA | -325.5 | 0.073 | | -682.0 | 31.1 |
| TIMP-1 | NJM | GJH | 3360.9 | 0.322 | | -3351.0 | 10072.7 |
|  | Age 18-29 | Age 30-49 | 1486.1 | 0.393 | | -1954.3 | 4926.6 |
|  |  | Age 50 + | 3589.7 | 0.178 | | -1661.9 | 8841.3 |
|  | Men | Women | 4914.6 | 0.325 | | -4957.6 | 14786.7 |
|  | DDwR | DDwoR | -194.7 | 0.957 | | -7435.9 | 7046.4 |
|  |  | DJD | -11094.1 | 0.000 | | -16392.3 | -5795.9 |
|  |  | CIA | -10357.9 | 0.000 | | -15720.7 | -4995.0 |
| TIMP-2 | NJM | GJH | -1266.1 | 0.750 | | -9138.6 | 6606.3 |
|  | Age 18-29 | Age 30-49 | -69.5 | 0.974 | | -4261.8 | 4122.7 |
|  |  | Age 50 + | -701.0 | 0.822 | | -6875.4 | 5473.3 |
|  | Men | Women | 5427.8 | 0.191 | | -2753.1 | 13608.7 |
|  | DDwR | DDwoR | 11674.0 | 0.003 | | 4000.3 | 19347.7 |
|  |  | DJD | -6654.0 | 0.079 | | -14094.8 | 786.7 |
|  |  | CIA | -8384.9 | 0.014 | | -14999.8 | -1770.0 |
| TIMP-3 | NJM | GJH | -507.9 | 0.618 | | -2524.6 | 1508.8 |
|  | Age 18-29 | Age 30-49 | 1423.8 | 0.234 | | -937.4 | 3784.9 |
|  |  | Age 50 + | 856.1 | 0.467 | | -1476.4 | 3188.7 |
|  | Men | Women | 2620.0 | 0.038 | | 142.3 | 5097.6 |
|  | DDwR | DDwoR | 1935.9 | 0.251 | | -1396.6 | 5268.5 |
|  |  | DJD | -3045.8 | 0.024 | | -5675.9 | -415.6 |
|  |  | CIA | -3224.6 | 0.025 | | -6040.5 | -408.7 |
| TIMP-4 | NJM | GJH | -21.8 | 0.252 | | -59.4 | 15.8 |
|  | Age 18-29 | Age 30-49 | 27.2 | 0.117 | | -6.9 | 61.3 |
|  |  | Age 50 + | -7.4 | 0.746 | | -52.8 | 38.0 |
|  | Men | Women | 25.0 | 0.249 | | -17.9 | 68.0 |
|  | DDwR | DDwoR | -20.5 | 0.283 | | -58.2 | 17.2 |
|  |  | DJD | -31.9 | 0.381 | | -103.9 | 40.2 |
|  |  | CIA | -32.7 | 0.174 | | -80.2 | 14.8 |
| Tenascin C | NJM | GJH | 4108.8 | 0.569 | | -10191.8 | 18409.3 |
|  | Age 18-29 | Age 30-49 | 6569.6 | 0.353 | | -7405.9 | 20545.2 |
|  |  | Age 50 + | 3619.7 | 0.548 | | -8313.0 | 15552.4 |
|  | Men | Women | -1566.2 | 0.813 | | -14695.6 | 11563.2 |
|  | DDwR | DDwoR | 9595.2 | 0.124 | | -2693.3 | 21883.7 |
|  |  | DJD | -3998.2 | 0.633 | | -20592.2 | 12595.8 |
|  |  | CIA | -3563.2 | 0.554 | | -15497.7 | 8371.2 |
| TREM1 | NJM | GJH | 111.3 | 0.571 | | -278.1 | 500.7 |
|  | Age 18-29 | Age 30-49 | 375.3 | 0.008 | | 99.5 | 651.1 |
|  |  | Age 50 + | 275.5 | 0.025 | | 34.8 | 516.1 |
|  | Men | Women | 144.6 | 0.578 | | -371.2 | 660.4 |
|  | DDwR | DDwoR | 111.3 | 0.550 | | -258.3 | 480.8 |
|  |  | DJD | 662.4 | 0.059 | | -26.8 | 1351.6 |
|  |  | CIA | 371.7 | 0.028 | | 40.6 | 702.8 |

CI, confidence interval; Coef., coefficient; CIA, chronic inflammatory arthritis; DDwR, disc displacement with reduction; DDwoR, disc displacement without reduction; DJD, degenerative joint disease; GJH, general joint hypermobility; NJM, normal joint mobility.
