# Supplementary material for: General joint hypermobility in temporomandibular joint disease; clinical characteristics, biomarkers, and surgical aspects
Source: Heliyon. 2023 Nov 30;9(12):e23051. doi: 10.1016/j.heliyon.2023.e23051 (PMC10750043; doi:10.1016/j.heliyon.2023.e23051)
Supplement: Multimedia component 2 [file mmc2.docx]

**Supplementary Table SII.** Multivariate quantile regression of ECM protein and ECM-related protein concentrations in patients in age group 18-28 with GJH or NJM.

| **Protein** | **Variables** | | **Coef.** | ***P*** | | **95% CI** | |
| --- | --- | --- | --- | --- | --- | --- | --- |
|  | ***Reference*** |  |  | |  | **Lower** | **Upper** |
| ADAMTS13 | NJM | GJH | -50800.0 | 0.789 | | -4.4x10^5^ | 3.4x10^5^ |
|  | Men | Women | 1.3x10^5^ | 0.524 | | -2.8x10^5^ | 5.3x10^5^ |
|  | DDwR | DDwoR | 2.9x10^5^ | 0.016 | | 60475.2 | 5.3x10^5^ |
|  |  | DJD | -1.2x10^5^ | 0.303 | | -3.6x10^5^ | 1.2x10^5^ |
|  |  | CIA | 55414.6 | 0.774 | | -3.4x10^5^ | 4.5x10^5^ |
| Aggrecan | NJM | GJH | 3026.2 | 0.713 | | -13800.0 | 19871.7 |
|  | Men | Women | -10400.0 | 0.757 | | -79500.0 | 58662.4 |
|  | DDwR | DDwoR | 5560.8 | 0.429 | | -8761.9 | 19883.4 |
|  |  | DJD | 22038.4 | 0.337 | | -24500.0 | 68567.8 |
|  |  | CIA | 27308.2 | 0.044 | | 779.9 | 53836.5 |
| Collagen1 α1 | NJM | GJH | 3285.0 | 0.874 | | -39300.0 | 45873.8 |
|  | Men | Women | -361.5 | 0.981 | | -31800.0 | 31088.2 |
|  | DDwR | DDwoR | 14612.5 | 0.246 | | -10800.0 | 40027.7 |
|  |  | DJD | 7379.6 | 0.641 | | -25000.0 | 39712.0 |
|  |  | CIA | -7124.5 | 0.469 | | -27200.0 | 12928.9 |
| Collagen4 α1 | NJM | GJH | -1236.5 | 0.733 | | -8646.7 | 6173.7 |
|  | Men | Women | -4525.1 | 0.550 | | -20000.0 | 10918.6 |
|  | DDwR | DDwoR | 6752.6 | 0.028 | | 785.4 | 12719.9 |
|  |  | DJD | -2776.5 | 0.477 | | -10700.0 | 5160.1 |
|  |  | CIA | 4387.9 | 0.795 | | -30200.0 | 38976.9 |
| FAP-α | NJM | GJH | 1803.6 | 0.567 | | -4632.2 | 8239.3 |
|  | Men | Women | 900.1 | 0.858 | | -9425.0 | 11225.2 |
|  | DDwR | DDwoR | 8480.1 | 0.087 | | -1351.1 | 18311.4 |
|  |  | DJD | 4546.0 | 0.503 | | -12500.0 | 6328.9 |
|  |  | CIA | 3810.4 | 0.391 | | -11200.0 | 4567.6 |
| Fibronectin | NJM | GJH | -1.7x10^5^ | 0.924 | | -3.8x10^6^ | 3.5x10^6^ |
|  | Men | Women | -2.6x10^5^ | 0.839 | | -2.9x10^5^ | 2.4x10^5^ |
|  | DDwR | DDwoR | -1.3x10^6^ | 0.107 | | -2.9x10^6^ | 3.1x10^5^ |
|  |  | DJD | -2.2x10^6^ | 0.010 | | -3.9x10^6^ | -6.0x10^5^ |
|  |  | CIA | -1.3x10^6^ | 0.770 | | -1.0x10^7^ | 7.5x10^6^ |
| HGF-r | NJM | GJH | 236.3 | 0.706 | | -1048.1 | 1520.7 |
|  | Men | Women | 1182.2 | 0.253 | | -908.2 | 3272.6 |
|  | DDwR | DDwoR | -580.2 | 0.502 | | -2344.6 | 1184.2 |
|  |  | DJD | -2469.1 | 0.027 | | -4633.3 | -305.0 |
|  |  | CIA | -1771.2 | 0.041 | | -3458.9 | -83.5 |
| ICAM-1 | NJM | GJH | 1.1x10^5^ | 0.661 | | -4.2x10^5^ | 6.5x10^5^ |
|  | Men | Women | -2.3x10^5^ | 0.520 | | -9.6x10^5^ | 5.0x10^5^ |
|  | DDwR | DDwoR | 6.2x10^5^ | 0.029 | | 67425.9 | 1.2x10^6^ |
|  |  | DJD | 60244.4 | 0.787 | | -4.0x10^5^ | 5.2x10^5^ |
|  |  | CIA | -30000.0 | 0.946 | | -9.3x10^5^ | 8.7x10^5^ |
| Lumican | NJM | GJH | -1.1x10^6^ | 0.445 | | -3.9x10^6^ | 1.8x10^6^ |
|  | Men | Women | 1.7x10^6^ | 0.447 | | -2.8x10^6^ | 6.1x10^6^ |
|  | DDwR | DDwoR | 2.9x10^6^ | 0.215 | | -1.8x10^6^ | 7.6x10^6^ |
|  |  | DJD | -1.3x10^6^ | 0.095 | | -2.9x10^6^ | 2.5x10^5^ |
|  |  | CIA | 3.9x10^5^ | 0.851 | | -3.9x10^6^ | 4.7x10^6^ |
| MMP-1 | NJM | GJH | 191.8 | 0.076 | | -23.0 | 406.5 |
|  | Men | Women | 70.3 | 0.621 | | -226.0 | 366.5 |
|  | DDwR | DDwoR | 184.7 | 0.122 | | -55.7 | 425.0 |
|  |  | DJD | Insufficient data | | | | |
|  |  | CIA | Insufficient data | | | | |
| MMP-2 | NJM | GJH | 18021.5 | 0.425 | | -28000.0 | 64055.3 |
|  | Men | Women | 18478.5 | 0.370 | | -23500.0 | 60445.7 |
|  | DDwR | DDwoR | -3673.9 | 0.854 | | -44800.0 | 37448.3 |
|  |  | DJD | -45600.0 | 0.018 | | -82700.0 | -8500.7 |
|  |  | CIA | -25100.0 | 0.362 | | -81200.0 | 30950.5 |
| MMP-7 | Insufficient data | | | | | | |
| MMP-9 | NJM | GJH | 6624.6 | 0.655 | | -23800.0 | 37052.2 |
|  | Men | Women | 9980.2 | 0.555 | | -24700.0 | 44619.3 |
|  | DDwR | DDwoR | -9072.3 | 0.591 | | -43700.0 | 25512.2 |
|  |  | DJD | -21300.0 | 0.372 | | -69900.0 | 27260.6 |
|  |  | CIA | -21500.0 | 0.321 | | -65400.0 | 22454.8 |
| MMP-10 | NJM | GJH | -25.4 | 0.939 | | -709.4 | 658.5 |
|  | Men | Women | 137.0 | 0.653 | | -489.4 | 763.4 |
|  | DDwR | DDwoR | 377.4 | 0.107 | | -88.5 | 843.3 |
|  |  | DJD | 1781.8 | 0.062 | | -102.0 | 3665.7 |
|  |  | CIA | 274.8 | 0.471 | | -504.5 | 1054.0 |
| NCAM-1 | NJM | GJH | -179.2 | 0.980 | | -14500.0 | 14157.4 |
|  | Men | Women | 6085.8 | 0.092 | | -1081.5 | 13253.2 |
|  | DDwR | DDwoR | 10800.2 | 0.019 | | 1990.7 | 19609.6 |
|  |  | DJD | -8699.2 | 0.734 | | -61200.0 | 43769.6 |
|  |  | CIA | -3950.7 | 0.470 | | -15100.0 | 7206.6 |
| OPG | NJM | GJH | -1156.8 | 0.818 | | -11500.0 | 9143.1 |
|  | Men | Women | 1143.7 | 0.935 | | -27500.0 | 29779.7 |
|  | DDwR | DDwoR | -9940.8 | 0.820 | | -99600.0 | 79685.1 |
|  |  | DJD | -9644.3 | 0.825 | | -99100.0 | 79856.2 |
|  |  | CIA | -11900.0 | 0.785 | | -1.0x10^5^ | 77292.1 |
| Osteonectin | NJM | GJH | 60964.8 | 0.581 | | -165000.0 | 287000.0 |
|  | Men | Women | 44369.3 | 0.635 | | -147000.0 | 236000.0 |
|  | DDwR | DDwoR | -11200.0 | 0.882 | | -166000.0 | 144000.0 |
|  |  | DJD | -1.6x10^5^ | 0.091 | | -3.4x10^5^ | 27072.6 |
|  |  | CIA | -74300.0 | 0.136 | | -1.7x10^5^ | 25333.2 |
| Syndecan-1 | NJM | GJH | 911.5 | 0.651 | | -3216.2 | 5039.3 |
|  | Men | Women | 311.8 | 0.759 | | -1771.3 | 2395.0 |
|  | DDwR | DDwoR | 749.6 | 0.656 | | -2688.5 | 4187.7 |
|  |  | DJD | 675.5 | 0.932 | | -15600.0 | 16981.9 |
|  |  | CIA | 5179.8 | 0.248 | | -3873.9 | 14233.5 |
| Syndecan-4 | NJM | GJH | 166.5 | 0.383 | | -220.5 | 553.6 |
|  | Men | Women | 355.5 | 0.025 | | 47.7 | 663.4 |
|  | DDwR | DDwoR | 65.3 | 0.728 | | -318.7 | 449.3 |
|  |  | DJD | -354.3 | 0.113 | | -799.4 | 90.8 |
|  |  | CIA | -307.8 | 0.260 | | -858.5 | 243.0 |
| TIMP-1 | NJM | GJH | -1431.5 | 0.836 | | -15700.0 | 12800.8 |
|  | Men | Women | 7070.4 | 0.298 | | -6707.1 | 20848.0 |
|  | DDwR | DDwoR | -2350.6 | 0.670 | | -13700.0 | 8971.5 |
|  |  | DJD | -10300.0 | 0.113 | | -23300.0 | 2660.2 |
|  |  | CIA | -11800.0 | 0.014 | | -20900.0 | -2656.1 |
| TIMP-2 | NJM | GJH | -4728.9 | 0.169 | | -11600.0 | 2175.1 |
|  | Men | Women | 8890.6 | 0.163 | | -3891.2 | 21672.3 |
|  | DDwR | DDwoR | 11674.0 | 0.005 | | 3954.5 | 19393.5 |
|  |  | DJD | -10100.0 | 0.005 | | -16800.0 | -3406.2 |
|  |  | CIA | -11700.0 | 0.015 | | -21000.0 | -2476.3 |
| TIMP-3 | NJM | GJH | -564.4 | 0.478 | | -2190.1 | 1061.3 |
|  | Men | Women | 2601.0 | 0.008 | | 739.3 | 4462.7 |
|  | DDwR | DDwoR | 1859.0 | 0.183 | | -945.1 | 4663.0 |
|  |  | DJD | -2142.0 | 0.063 | | -4410.9 | 126.9 |
|  |  | CIA | -2735.0 | 0.004 | | -4493.1 | -976.9 |
| TIMP-4 | NJM | GJH | -10.4 | 0.916 | | -213.4 | 192.6 |
|  | Men | Women | 45.2 | 0.325 | | -48.1 | 138.6 |
|  | DDwR | DDwoR | -55.7 | 0.321 | | -169.8 | 58.4 |
|  |  | DJD | 366.6 | 0.080 | | -47.3 | 780.6 |
|  |  | CIA | -31.3 | 0.848 | | -367.5 | 304.9 |
| Tenascin C | NJM | GJH | -128.1 | 0.991 | | -24600.0 | 24386.8 |
|  | Men | Women | -633.4 | 0.977 | | -44900.0 | 43658.6 |
|  | DDwR | DDwoR | 4937.1 | 0.700 | | -21300.0 | 31152.3 |
|  |  | DJD | -9537.4 | 0.513 | | -39300.0 | 20219.01 |
|  |  | CIA | -8221.3 | 0.522 | | -34400.0 | 17972.3 |
| TREM1 | NJM | GJH | 111.3 | 0.804 | | -815.5 | 1038.0 |
|  | Men | Women | -326.9 | 0.428 | | -1171.0 | 517.2 |
|  | DDwR | DDwoR | 36.8 | 0.872 | | -436.5 | 510.2 |
|  |  | DJD | 1981.7 | 0.040 | | 104.8 | 3858.6 |
|  |  | CIA | 371.7 | 0.580 | | -1011.7 | 1755.1 |

CI, confidence interval; Coef., coefficient; CIA, chronic inflammatory arthritis; DDwR, disc displacement with reduction; DDwoR, disc displacement without reduction; DJD, degenerative joint disease; GJH, general joint hypermobility; NJM, normal joint mobility.
